# Severe Illness Anxiety Treated by Integrating Inpatient Psychotherapy With Medical Care and Minimizing Reassurance

**DOI:** 10.3389/fpsyt.2019.00150

**Published:** 2019-03-22

**Authors:** Albert T. Higgins-Chen, Sarah B. Abdallah, Jennifer B. Dwyer, Alfred P. Kaye, Gustavo A. Angarita, Michael H. Bloch

**Affiliations:** ^1^Department of Psychiatry, Yale School of Medicine, Yale University, New Haven, CT, United States; ^2^Yale School of Medicine, Yale University, New Haven, CT, United States; ^3^Child Study Center, Yale School of Medicine, Yale University, New Haven, CT, United States; ^4^Veterans Administration National Center for PTSD, West Haven, CT, United States

**Keywords:** illness anxiety disorder, health anxiety, hypochondriasis, panic disorder, psychotherapy, CBT (cognitive-behavioral therapy), case report

## Abstract

Illness anxiety disorder (IAD, formerly hypochondriasis) is characterized by preoccupation with fear of serious illness despite medical reassurance. IAD is common, debilitating, challenging to treat, and results in high healthcare utilization. Outpatient management of IAD is challenging because patients can compulsively seek reassurance from numerous providers, which interferes with learning more productive coping skills. We present the case of a woman with severe IAD who presented to the emergency room with increasing frequency over several months, despite regular outpatient medical visits and escalating psychiatric care. We made the unusual decision to hospitalize her for IAD for 1 month, in the absence of typical hospitalization criteria. This hospitalization allowed us to consolidate all medical and psychiatric care into a single provider team and train all staff and family to communicate with her in a consistent manner. We successfully treated her by integrating a general cognitive-behavioral therapy (CBT) protocol into medical care and decision-making. In response to her numerous health concerns, we minimized medical work-up, reassurance, and reactive medication changes, and instead used the concerns as opportunities to reinforce the psychotherapy. This approach allowed us to simplify her medication regimen and manage her co-morbid hypertension and vitamin deficiencies. Though inpatient hospitalization is likely infeasible in most cases of IAD, outpatient providers may create similar treatment plans based on the example of our case report, without needing highly specialized expertise. Such a plan would require a straightforward understanding of IAD psychology, which we review here, combined with readily accessible tools including a universal CBT protocol, online CBT courses, and clinical symptom scales. We discuss our approach for responding to health concerns, maintaining therapeutic alliance, integrating CBT principles into patient interactions, and managing medications. Since patients with IAD share health concerns with all providers, staff, and family, we also include our own IAD communication guide, appropriate for a general audience, that demonstrates how to respond in these conversations.

## Introduction

Illness anxiety disorder (IAD) is characterized by excessive concern about acquiring or having serious medical illnesses that interferes with normal functioning, persisting despite normal medical workup and reassurance. Patients with IAD may seek excessive medical care or request frequent changes to medical care (care-seeking type), or may avoid medical care, fearing the possibility of true disease, or adverse effects of treatment (care-avoidant type). IAD and somatic symptom disorder are DSM-5 diagnoses that correspond best to hypochondriasis in DSM-IV. Illness anxiety is common, with studies reporting lifetime prevalence of 2–13% in the general population, and 5–30% in patients with comorbid medical conditions (1, 2). Previous experiences with illness, especially those associated with strongly negative emotions can contribute to anxiety disorders including IAD (3, 4). Severe IAD often results in high healthcare utilization and is unlikely to resolve sporadically (5). Thus, all physicians should be prepared to work with IAD patients and refer them for proper treatment.

Well-intentioned physicians may try repeatedly reassuring patients or changing medical management to allay patients' anxieties, but unfortunately this is counter-productive in the absence of concurrent psychotherapy. According to cognitive-behavioral models that form the basis for effective evidence-based treatment for IAD (6), the relief associated with reassurance makes the patient more likely to seek reassurance in response to future health concerns. Similarly, changing medical management based solely on patient anxiety only results in worse anxiety to the new plan. Both actions distract from psychotherapeutic interventions targeting underlying cognitive biases that generated anxiety in the first place. Many generalized cognitive-behavioral therapy (CBT) protocols may be effectively applied to IAD (2). However, the primary challenge for IAD treatment is integrating CBT with medical care. Our case highlights this challenge and can serve as a guide for any outpatient provider to integrate readily available resources to treat IAD.

## Case Report

We present a woman in her 40's who was never married, lived with mother, boyfriend, and three children, and was unemployed and on Social Security disability. She had a 20-year history of generalized anxiety and major depression that was in remission for the past 5 years with outpatient therapy, escitalopram 40 mg, and lamotrigine 100 mg daily. Medical conditions included hypertension, long-standing iron deficiency anemia, a 30 pack-year history of tobacco use, but no alcohol or substance use. She had faced numerous health problems in various family members over the past 2 years, most notably her brother's sudden unexpected death shortly after doctors told him he was in remission from a serious illness. Other family diagnoses included cancer, stroke, retinal detachment, bipolar disorder, and multiple suicides.

The patient developed severe illness anxiety shortly after her son's ICU hospitalization for influenza. She ruminated about health, worried that she or her loved ones would die, and researched symptoms and medications on the Internet for over 4 hours each day. She began presenting to the emergency room (ER) for normal physical sensations and anxiety-related symptoms, and each time she was reassured by a normal medical workup. Over the next 3 months, the frequency of ER visits steadily increased despite escalating psychiatric care, medication changes, and regular outpatient medical visits. She started aripiprazole but self-discontinued it, believing it caused a dizzy spell and a globus sensation, even though these symptoms began before the aripiprazole trial. The globus sensation caused a fear of choking for 2 weeks, so she ate very little and lost 20 pounds. One poor-quality EKG in the ER showed prolonged QTc of 490, prompting her to call her primary medical doctor (PMD) daily for a week despite subsequent normal EKGs. She entered an intensive outpatient program (IOP), which attempted to trial various medications over 2 months while tapering lamotrigine and escitalopram. She also had her first inpatient psychiatric hospitalization for 3 days for passive suicidal ideation. Clomipramine was discussed for possible obsessive-compulsive disorder (OCD). However, each time a new medication was started she read about side effects, believed she had them, and presented to the ER.

Four months into her IAD course, her PMD measured a blood pressure of 210/120 after she had stopped her lisinopril and hydrochlorothiazide for 2 months from fear of side effects. She had no symptoms and resumed her antihypertensives. However, she began checking her blood pressure 5 or more times per day, constantly worried about having a stroke, and presented to the ER ten times in 1 week. Given she had utilized so many emergency resources, she was considered for standard psychiatric hospitalization but did not meet criteria. Our inpatient unit was able to offer intensive psychotherapy, so she was transferred to us.

On admission, she reported her mood as anxious and depressed, with no suicidal ideation. Scales administered for self-reported symptoms indicated severe IAD (Table 1). They also indicated moderate depression and severe panic, but her presentation was more consistent with IAD. We also considered generalized anxiety disorder (GAD), but her ruminations focused primarily on health and not on other topics. The only lifetime symptom on a Y-BOCS symptom inventory for OCD was “concern with illness or disease” so we did not complete a Y-BOCS scale, as any findings would be more consistent with IAD. Laboratory studies were notable for iron-deficiency anemia (Hgb 8.6, ferritin 3, at her baseline), low vitamin D, and borderline-low vitamin B12. For these long-standing deficiencies we tested transglutaminase IgG/IgA levels but they did not indicate celiac disease. MMSE was 30/30.

**Table 1:** Patient's scores on clinical symptom scales over time.

**Location**	**Admission**	**Discharge**	**Outpatient**	**IOP**	**IOP**	**IOP**	**Outpatient**
Time (weeks)	0	4	6	7	10	15	24
SHAI (healthy anxiety)	41	45	35	27	-	-	10
PHQ-9 (depression)	19	8	7	7	5	7	4
PDSS-SR9 (panic)	19	-	-	-	7	4	2

*SHAI, Short Health Anxiety Inventory; maximum 54; score > 27 supports diagnosis of IAD over other anxiety disorders. PHQ-9, Patient Health Questionnaire for depression; maximum 27; score > 20 supports diagnosis of severe depression, 15–19 moderately severe, 10–14 moderate, 5–9 mild, 0–4 minimal. PDSS, Panic Disorder Severity Scale; maximum 28; score > 14 supports diagnosis of severe panic, 10–13 moderate, 6–9 mild, 2–5 minimal. IOP, intensive outpatient program. We also performed a Y-BOCS symptom inventory which did not find evidence for current or past obsessive-compulsive disorder*.

We diagnosed the patient with IAD with minimal somatic symptoms. Given comorbid depression, we opted for CBT using the Unified Protocol for Transdiagnostic Treatment of Emotional Disorders (UP) (7). We completed 12 CBT sessions, three times per week for up to 90 min each, at a rate of about one UP workbook chapter per session. We provide UP worksheets to record her thoughts, feelings, sensations, and behaviors in different contexts. UP chapters addressed: (1) motivations for change, (2) reducing cognitive distortions by recognizing and correcting automatic negative appraisals of situations as well as weighing evidence, (3) increasing distress tolerance through mindfulness and non-judgmental awareness of emotions, (4) reducing emotional avoidance, and (5) exposure therapy to anxiety-provoking stimuli.

**Figure 1:**
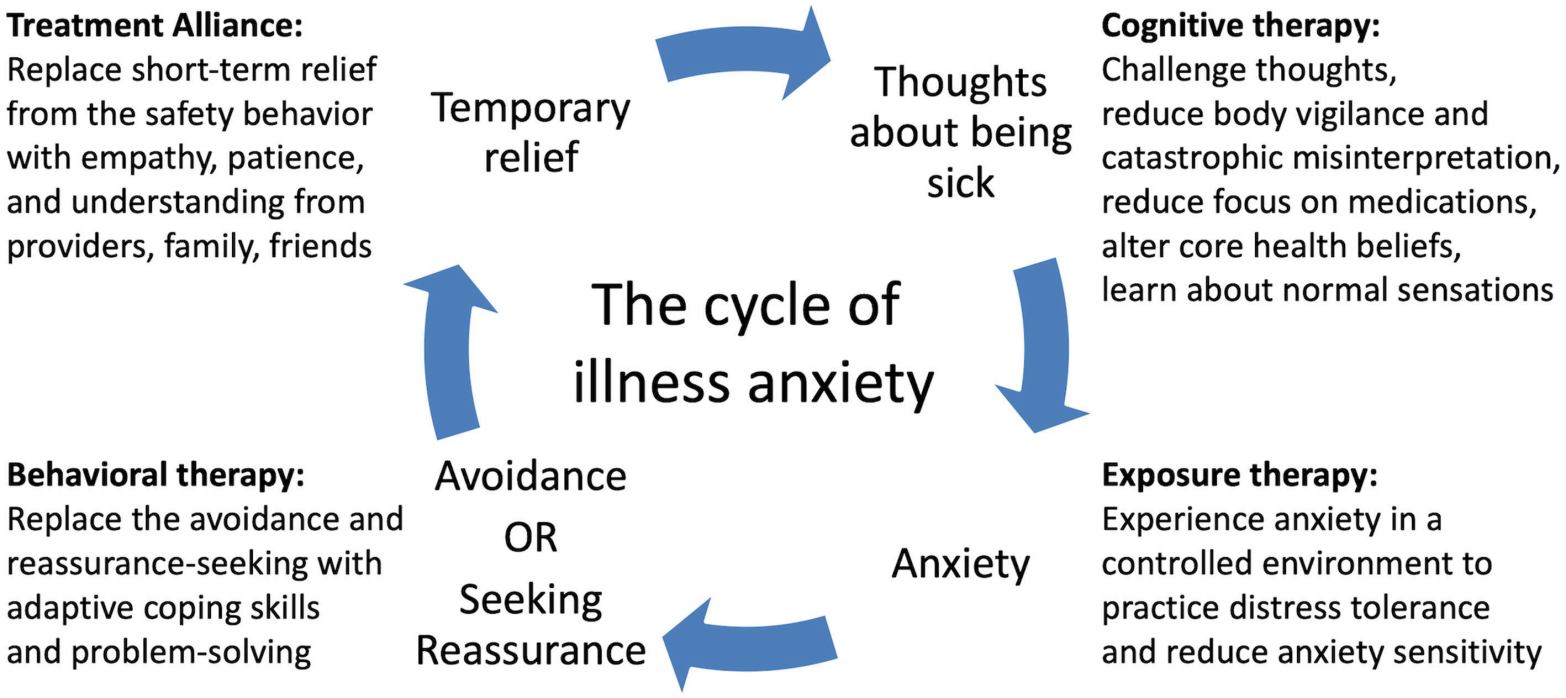
A cognitive-behavioral model of illness anxiety. A feedback loop of thoughts of being sick and safety behaviors (avoidance or reassurance-seeking) causes worsening of illness anxiety over time. Cognitive, exposure, and behavioral therapies as well as strong therapeutic alliance can break the cycle and improve symptoms.

We also introduced a simple cognitive model of illness anxiety (Figure 1): her fears of being sick or experiencing serious medication side effects made her anxious, so she either avoided the problem (e.g., by refusing medications) or sought reassurance, and the reward of her short-term relief only made her original thoughts stronger. She realized that to break this cycle, she needed to replace reassurance-seeking with other coping skills and expose herself to the anxiety-provoking stimuli she was avoiding, and this realization allowed her to better engage in therapy.

Importantly, this psychotherapy used a readily accessible protocol and did not involve specialized CBT for IAD, as multiple generalized forms of CBT are effective for IAD (2). The key advance of this case was to incorporate psychotherapeutic principles into medical care and decision-making. To that end, we encouraged the patient to apply the IAD cycle and the CBT concepts during any conversation about medical care. For example, we had opted to simplify her medication regimen as much as possible, as we felt over-fixation on medications in the outpatient setting had worsened her illness anxiety and distracted from psychotherapy. We established a plan early on to increase her mid-taper escitalopram dose from 15 to 20 mg, finish her lamotrigine taper, increase her antihypertensives and start iron and vitamin supplements. As expected, she expressed numerous concerns about the changes, often changed her mind about what she wanted and why, frequently researched side effects on the computer, and often refused changes. We recognized these behaviors were manifestations of avoidance common in anxiety disorders, and thus changing the plan based on her concerns would worsen her anxiety in the long term. We responded by having long, patient conversations with her about why the plan would help her and made very few changes to it. We frequently redirected her attention to the IAD cycle, and eventually she realized the primary problem was her anxiety rather than valid concerns about side effects.

The medications also became a form of exposure therapy. For example, when she had fears that a vitamin B12 injection would cause anaphylaxis, we incorporated it into a therapy session by asking her to describe her emotional experience and remind herself of the IAD cycle while receiving the injection. She feared the lamotrigine taper would cause a seizure, so she asked us numerous times to delay the taper and sought reassurance for weeks after it was stopped. For psychotherapy purposes, we did delay a dose reduction by 3 days and asked her to notice the result. She had predicted this would reduce her anxiety, but it actually caused far greater anxiety over those 3 days as she awaited the eventual dose change. In later therapy sessions, she developed the insight that this was an example of unhelpful emotional avoidance. Ultimately, she finished her lamotrigine taper and received escitalopram 20 mg and vitamins B12 and D. However, she did not consistently take iron or the increased antihypertensive dose during the admission.

We also had her apply the IAD cycle when she approached us multiple times per day about various minor symptoms and normal physical sensations (tight muscles, pinching sensations, sweaty palms, etc.). While we addressed any possibly emergent medical concerns, we deliberately avoided immediate clinical assessments and reassurance for most benign symptoms. Instead, we calmly asked her to apply psychotherapy principles to each concern, so she eventually realized that any reassurance-seeking would only bring temporary relief and worsen her anxiety in the long term. Psychoeducation was provided on anxiety symptoms and on how to manage symptoms in a non-medical manner (e.g., tennis ball self-massage for tense muscles). Several times she expressed frustration that her medical concerns were not addressed, stating, “Psychiatric patients can have medical problems too!” We maintained the therapeutic alliance through self-disclosure: we told her that as physicians, we did feel uncomfortable not immediately addressing her medical concerns but employed this communication strategy because it was in her best interest and we wanted to help her recover.

The patient regularly raised her health concerns with all staff on the unit and her family throughout her hospitalization. Thus, we implemented multiple psychoeducation sessions for staff and family so they could understand why IAD was driving the patient to seek reassurance, avoid getting frustrated with the patient, and respond appropriately. Furthermore, we created a written communication guide that includes specific scenarios and responses along with CBT principles they could discuss with the patient. We provide a modified copy here in the Supplementary Information.

For continuing care after discharge, we sent the communication guide and UP to outpatient psychiatric and medical providers to continue the management strategy that worked well in the inpatient setting. We also prescribed an online course specifically designed for health anxiety through the website ThisWayUp (https://thiswayup.org.au/). We suggested further ThisWayUp courses on panic, depression, and stress management which she completed over 3 months after discharge. A visiting nurse would administer her medications to provide continuing exposure therapy and ensure her hypertension was adequately addressed. Though she had significant anxiety about leaving the hospital, she discharged home without difficulty after 1 month.

Though she still met criteria for severe IAD at discharge (Table 1), this was expected given that 3 months of psychotherapy are needed to observe clear benefits of CBT (2, 8). Nevertheless, she had learned skills that allowed her to better function with her anxiety in the community without regularly presenting to the ER. We followed up with the patient 4 months after discharge, after she had finished her treatment at IOP level of care and the ThisWayUp online course. Though she still had some anxiety, she no longer met criteria for IAD (Table 1), spent almost no time on Internet research, and was able to address a family member's surgery without significant anxiety. She received continued care for severe hypertension, vitamin deficiency, and anemia, including with intravenous iron. She made a total of 4 ER visits over 4 months, substantially lower than the 10 visits in 1 week prior to admission.

## Discussion

Our case demonstrates the utility of integrating psychotherapy with medical care in treating IAD, and many aspects of our management can be replicated by outpatient medical providers. Measurement-based care through clinical symptom scales can help provide the proper diagnosis. For example, the cardinal symptoms and underlying psychology behind OCD, panic disorder, and IAD overlap significantly, but clinical scales can adequately distinguish between them (9). We used the 18-item Short Health Anxiety Inventory (SHAI; 0–3 scale for each item). Various cutoff scores for IAD have been utilized, ranging from 15 to 38, with 27 providing balance between sensitivity and specificity when differentiating between IAD and other anxiety disorders (10).

Cognitive behavioral therapy (CBT) is the mainstay of treatment for IAD. No CBT subtype has yet proven superior to our knowledge. Several studies support CBT (8, 11, 12) or specific subtypes, including cognitive (13), exposure response (13), group (14), or mindfulness CBT (15). In the outpatient setting, therapy may take 3 or more months to demonstrate improvements over standard care, but the benefits usually persist even at 5-year follow-up (2, 8). Online CBT for health anxiety and comorbid depression is an effective supplement or alternative to in-person psychotherapy (16, 17). Online courses such as ThisWayUp (https://thiswayup.org.au/) have the additional benefit of incorporating clinical symptom scales reported to the prescribing physician.

Medications can supplement IAD treatment. Randomized controlled trials support efficacy of SSRIs in treating IAD though they are less effective than psychotherapy (18, 19). Case reports suggest TCAs such as clomipramine may also be helpful (6). However, IAD patients often prefer and tolerate psychological treatment better than pharmacological treatment (20). Providers should be aware that excessive focus on medications to treat IAD may actually worsen the anxiety.

While most medical providers may not provide dedicated psychotherapy, they can help reinforce CBT skills in the setting of medical decision-making if they are familiar with the concepts. The model we taught to our patient, family, and staff illustrates the core mechanism of IAD and how CBT targets different features of IAD (Figure 1). IAD worsens over time because of the self-reinforcing cycle of anxiety and safety behaviors. An obsessional thought or fear of illness causes anxiety, which prompts the patient to either seek reassurance or avoid the issue. The temporary relief of the safety behavior is rewarding and strengthens the original obsessional thought and automatic behavior while also preventing the patient from realizing the fear was unfounded in the first place. Specific cognitive biases make patients particularly vulnerable to this cycle (9). Increased intolerance of uncertainty drives patients to seek out expert opinion even if there is a negligible chance of disease. Increased body vigilance and catastrophic misinterpretation causes them to constantly scan their bodies and find evidence for serious illness. Core beliefs about health may be erroneous, e.g., “health means having no physical or mental symptoms at all.” (6) Psychotherapy systematically modifies these thoughts and behaviors.

The key advance of this case report is illustrating how to help patients apply CBT concepts in the context of real-world medical decision-making. Instead of offering immediate reassurance or reactive medication changes, we used a set of communication strategies to encourage the patient to employ CBT in response to all health concerns. Of note, patients with IAD raise health concerns with various medical providers, staff, and families. Based on our experience, all these individuals can be recruited to help the patient apply CBT assuming they receive proper psychoeducation. Thus, we present a communication guide (Supplementary Information), appropriate for a general audience, that provides psychoeducation and communication strategies for everyone involved in the patient's care.

Integration of psychotherapy and medical care might be replicated in the outpatient setting by regular check-ins with psychiatric and medical providers who are in frequent communication with each other. In integrated primary care settings, providers with psychotherapy training could consult on cases and join medical visits with the primary physician. A plan should be developed between outpatient providers and the patient to avoid excessive ER visits. Nurses may be more effective than psychologists at engaging patients in IAD treatment (21), as they can discuss both the psychological and medical issues. A visiting nurse can administer medications as a form of exposure therapy and reduce IAD's interference with important medical care.

Hospitalizing patients for IAD alone is likely not an option for most cases in the current healthcare system. However, in severe cases of IAD, it can be extremely difficult in the outpatient setting to prevent excessive medical care-seeking, and each time the patient receives reassurance this may reinforce maladaptive behavior. We observed that hospitalization limits the patient to a single combined team providing psychiatric and medical care, thus preventing reassurance-seeking from various ER providers and providing an opportunity for extinction of the maladaptive behavior. This is reminiscent of eating disorder treatment, where inpatient and partial hospitalization programs can impose limits on eating behaviors long enough for CBT to take effect. We suggest similar programs for IAD can be explored. In the future, such programs might not necessarily require a full month; a recent paper on a novel 4-day intensive protocol for OCD showed sustained remission for 4 years in 56 of 77 patients (22). New treatment options for IAD are needed in modern healthcare systems, where psychotherapy and medical care are usually delivered by separate providers with very different training. This is problematic as patients may not be empowered to apply psychotherapeutic principles in the same setting where they are making healthcare decisions, and innovations in healthcare delivery are needed to address this issue.

In conclusion, evidence-based CBT is highly effective for the treatment of IAD, but a major challenge is integrating it into medical care. Though most physicians do not directly provide psychotherapy, they can care for patients by understanding the underlying psychology of IAD, helping patients apply CBT principles during medical decision-making, avoiding excessive reassurance or medication changes, and educating all staff and family about how to respond to patients' health concerns.

## Data Availability

All datasets generated for this study are included in the manuscript and/or the Supplementary Files.

## Ethics Statement

This study was performed in accordance with the provisions of the Declaration of Helsinki and the Yale University Human Subjects Committee. We obtained written informed consent from the patient authorizing publication of the clinical case. She read the case report before publication and her anonymity has been preserved.

## Author Contributions

AH-C and SA served as primary physicians for the patient during the hospitalization, collected and analyzed the data, and wrote the manuscript. JD, AK, GA, and MB provided supervision for care of the patient and reviewed the manuscript.

### Conflict of Interest Statement

The authors declare that the research was conducted in the absence of any commercial or financial relationships that could be construed as a potential conflict of interest.
