# Supplementary material for: Severe Illness Anxiety Treated by Integrating Inpatient Psychotherapy With Medical Care and Minimizing Reassurance
Source: Front Psychiatry. 2019 Mar 22;10:150. doi: 10.3389/fpsyt.2019.00150 (PMC6438952; doi:10.3389/fpsyt.2019.00150)
Supplement: Supplementary file 1 [file Table_1.DOCX]

Illness Anxiety Disorder:

A Communication Guide for Families and Medical Providers

*Disclaimer: This document is a general educational resource on helping people with illness anxiety disorder diagnosed by a physician. The guidelines here may not apply to every case. Healthcare providers who distribute this to staff, patients and families are responsible for ensuring the material presented here is accurate and appropriate for their patients’ care. This guide does not constitute a medical or psychological treatment.*

# What is Illness Anxiety Disorder (IAD)?

Illness anxiety disorder (IAD), also known as health anxiety or hypochondriasis, is excessive worry about having or developing a serious illness despite reassurance from a medical provider. While it is normal to have some health concerns, IAD is well outside the range of normal worry, often seeming extreme to others. A person with the disorder usually will have few or no physical symptoms, and a thorough medical exam will not show any signs of the illness(es) they are worried about. However, their disorder might make them interpret minor symptoms (such as a headache or small aches and pains) as signs they have a serious illness. Symptoms can worsen after major stressors, especially those involving the health of loved ones.

**Regardless of whether physical symptoms are present, the anxiety itself is causing severe distress and should be taken seriously.** IAD can worsen to the point where people spend most of their day doing things that take away their anxiety temporarily (such as going to the doctor, looking up medical information on the internet, or checking their bodies for signs of disease). They may also avoid things that make them anxious. For example, they may avoid necessary medications due to excessive fear of side effects or avoid driving out of fear of having a medical emergency while driving. This avoidance makes them feel better temporarily but only makes them more likely to be anxious about future situations. When a person’s illness anxiety is bad enough, it can interfere with their job, social life, and ability to care for themselves and others.

# How can I help someone with IAD?

It is emotionally difficult to see someone you care about struggle with IAD. Fortunately, you can help. Every time a person with IAD approaches someone else with a health concern, that is an opportunity to help the person. **However, you must first learn how to respond to health concerns properly, based on a correct understanding of the psychology of IAD.**

A highly effective treatment for IAD is a form of psychological counseling called cognitive behavioral therapy, or CBT. CBT gives people new skills to examine and deliberately change their own thoughts and actions. However, when a person is terrified by the health fears of IAD, it is difficult to remember what one has learned in CBT. **Families, friends, and medical providers can all help the person apply CBT to their health concerns.**

There are three big ways you can help a person with IAD:

1. **Learn the psychology of IAD** to understand what they are going through
2. **Support them in applying CBT concepts to medical decisions,** but don’t force them to change or tell them what to think.
3. **Patience and empathy.** Know they have a real condition and they are doing the best they can to recover. Make them feel supported regardless of how severe the IAD is.

# What causes IAD? Why does a person with IAD do what they do?

It’s important to remember that it is completely normal to have some health concerns. Everyone pays attention to their bodies, learns what is normal, and learns what is potentially dangerous. In IAD, this normal learning process goes awry. It is not clear what causes this, and there are probably many possible causes. Sometimes IAD might be triggered or worsened by an extremely stressful experience involving serious illness in a family member. This might focus a person’s thinking excessively on health, and the extreme stress makes proper learning more difficult.

**Once illness anxiety starts, it may be sustained and worsened over time by** **maladaptive actions that are** **meant to relieve short-term anxiety but unfortunately worsen long-term anxiety**. The first maladaptive action is reassurance-seeking: for example, a person with IAD may seek reassurance from emergency doctors for a minor physical sensation. The second maladaptive action is avoidance: for example, a person may avoid taking a medication that they need because their mind tells them it is poison. Both these actions (reassurance-seeking and avoidance) bring temporary relief. However, this temporary relief is rewarding, which causes the initial worry to come back even stronger next time. IAD can take on a life of its own, continuing long after the initial trigger. See the figure below to understand **the IAD cycle**.


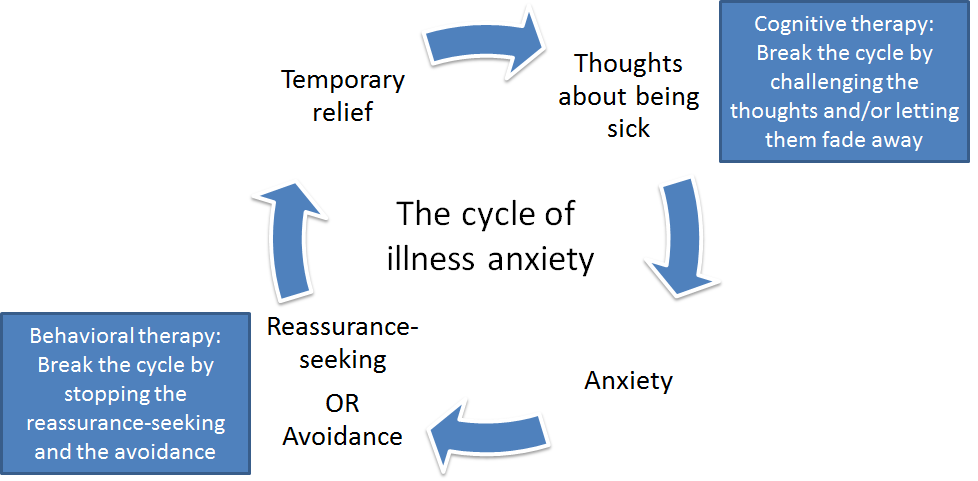


“Observe your thoughts until they fade away.”

“The best way to address anxiety is to do the thing you are anxious about doing.”

# Five big CBT ideas for IAD

**You don’t need to understand everything about CBT to support a person with IAD. You just need to know the big ideas, and then ask the person with IAD to apply the CBT concepts whenever they have health-related fears.**

1. **Remind yourself:** **The real problem is the thought or fear** that a physical symptom is dangerous. The problem is not the physical symptom itself. Visualize the IAD cycle (previous page).
2. **Your thoughts are not necessarily true:** There are many “thinking traps” that can lead one to believe erroneous things. Respond to health fears either by **weighing the evidence** that the fear is true or by simply observe the thought **until it fades away**.
3. **Do not seek or provide immediate reassurance for health fears**: Use the fears as an opportunity to practice CBT principles first (see section 5 for examples). Delay the reassurance as long as possible.
4. **Turns towards anxiety, not away from it:** If you are anxious about something (going to a specific place, taking specific medications), **then the best cure for the anxiety is to *do the thing you are anxious about.*** The more difficult it is, the more it will help.
5. **Learning takes time:** Each time you practice CBT, you get a little bit better. Keep practicing until it becomes a habit to examine and change your thoughts, a habit to delay reassurance, and a habit to do the thing you are anxious about. If it took you 3 months to develop IAD, it may take 3 months to fully undo the IAD.

# How do I respond to health concerns from a person with IAD?

Remember, people with IAD will look for reassurance that they are okay from others. **Providing excessive reassurance can make them feel better in the short-term but will worsen their anxiety and need for reassurance in the long-term.** There may be many times where reassurance is necessary, but you have to **pair the reassurance with CBT ideas.**

1. **Acknowledge** that they are having a difficult time and support them emotionally.
2. **Take a deep breath and pause.** They may approach you frantically as if it is an emergency. Taking the time to respond thoughtfully can itself be helpful.
3. **Be curious and try to understand their experience.**
4. **Ask them to think** about the anxiety using CBT concepts and the IAD cycle. Ask them what they have learned in psychotherapy sessions and how they might apply it to their current fears.
5. **Ask them what they plan to do** based on this thinking. It’s important to empower them to come up with the plan, not someone else.
6. **Roll with resistance.** They may struggle to apply CBT concepts or become frustrated that you aren’t offering reassurance. Just remain calm, accept their frustration, and keep trying different ways to guide them to using CBT.
7. **Offer advice sparingly and only with permission.** Resist the urge to “tell them the right answer.” They have to apply the CBT concepts themselves in order to get better.
8. **Be patient.** You may experience thoughts that the person with IAD is deliberately being difficult and frustrating, or not trying hard enough. Setbacks may be common, and it may take months to recover. **Remind yourself of the psychology of IAD and how distressing it is to the person struggling with IAD. Seek support for yourself as well.**

# Examples of responding to health concerns in IAD

## “I have [insert symptom], I’m in danger, I might have [insert disease].”

- **Be curious and try to understand their internal experience**. What is their fear? What triggered it? What do they feel in their body? Does it fit into the IAD cycle? When they answer these questions, they self-reflect, which is helpful.
- **Ask them to look at the evidence for and against their fear**. If this is something they experienced in the past, think of what the outcome was. If it is something new think about the probability it is actually something serious.
- **Ask them to identify their response to their fear** and ask them if they think it is beneficial. Don’t immediately point out that they are seeking reassurance thus perpetuating the IAD cycle, rather let them come to their own conclusion. Ask them how else they could respond to the fear.
- **Wait and observe.** Ask them to observe their fear and what they feel, without judging it. Support them in doing this until the fear subsides.
- **Be open to the possibility that they actually do have a dangerous symptom**. First, you don’t want to miss a truly dangerous condition. Second, if you simply dismiss their concerns, it only creates conflict, which is not helpful. However, for a person with IAD, the probability any given symptom is actually dangerous is much lower than they think.

## “You’re ignoring my medical problems” or “You don’t care about me.” (in response to not offering immediate reassurance)

- **Do not take this personally.** Their fear and need for reassurance are so strong that they may say hurtful things. Don’t become defensive or say, “But I’m just trying to help you!”
- **You can respond to build trust**: “How do you think I feel about how I’m responding to you right now?” Then listen carefully and think about their response. What to say next depends on what they say, but your goal is to build trust. If you are a healthcare provider, you can say something like, “I do feel uncomfortable because as a doctor I’m trained to take medical complaints very seriously, but I’m communicating with you like this because it’s in your best interest and I want to see you recover.” If you are family or friend, you can say, “It feels bad to see you suffering and I want to relieve your short-term anxiety, but I also want you to recover in the long-term.”

## “But I have real medical problems too! I can't ignore them!”

- **Acknowledge** that everyone gets medical problems, including those with IAD.
- **Ask** them if they think their response to their symptoms or their medical issues is helpful
- **Discuss** that the point is not to ignore all medical problems. Instead, the point is to learn to address medical problems more effectively, and not get distracted by minor physical sensations or concerns.

## "I need to go to the emergency room."

## Understand why they want to go and ask them to reason if it is appropriate

## Ask them about alternative actions they could take. You could suggest watchful waiting, taking just 15 minutes to look up health information, making an appointment with a primary care physician, etc.

## Consider if the person truly does need to go to the emergency room. If you think this is possible, try to contact the primary care physician first. In the long term, work with a primary care physician to make a plan about when you would go to the emergency room and stick to that plan as best you can.

## "I don’t want to take this medication.” (or make a certain medication change)

- **Be curious and try to understand why**. What is their fear? Is it about a side effect? What triggered the fear? Does it fit into the IAD cycle? When they answer these questions, they self-reflect which is helpful. They may not even realize what their fear is, so be patient.
- **If you find a fear, ask them to look at the evidence for and against their fear.** It may be helpful to ask what health professionals or other reliable sources have told them. If they read Internet stories about side effects, ask them if they think they can trust those stories. This also is a good time to help them identify **thinking traps** (thinking the worst, jumping to conclusions) that are preventing them from approaching a situation logically.
- **Avoidance in the IAD cycle:** Point out that avoidance may be a big part of why they don’t want to take the medication. Ask them if this is part of the IAD cycle. Discuss that if you let them avoid the medication, **that may worsen their anxiety in the long term.**
- **Minimize medication changes.** Pick a medication plan and stick with it, only making changes as absolutely necessary. Remind them that making medication changes based solely on anxiety will only make the anxiety worse in the long term.
- **Notice if they are constantly changing their mind about what they want.** This is common with avoidance because they develop fear about whatever the current plan is. Ask them if they have noticed this too.

## “I’m not on the right medications for anxiety, that’s why I’m anxious.”

- **Discuss the following:** CBT is the treatment for IAD. Some medications might help in the long term by increasing a person’s ability to engage in CBT. Others may temporarily relieve the anxiety but will not be helpful in the long term. Speak to a physician about whether medications would be helpful.
- **Do not attribute improvement or worsening of illness anxiety solely to medications.** Focusing too much on medications may provoke a person with IAD to worry more about side effects or being on the “right” regimen, which only worsens the illness anxiety.

## "I don't want to feel this way anymore."

- **Express empathy.** Let them understand you realize how difficult this is and take their feelings seriously.
- **Understand emotions.** Ask them if they can identify specific thoughts and feelings that are making them feel worse right now. Ask if there were any events that are contributing to their negative emotions.
- **Problem-solve.** Is there a specific problem that you can address or start to work on right away? If so, talk with them about ways to address it.
- **Nonjudgmental awareness.** Instead of dwelling on the negative emotions, you can use this as an opportunity to practice nonjudgmental awareness. Ask them to observe their emotions and allow them to exist without judging it.
- **Encourage patience.** Remind them that treating IAD is a learning process to discover that medications and physical sensations are not dangerous (or nowhere near as dangerous as they thought). All learning takes time. It took months to develop illness anxiety, so it may take months to un-do it.
- **Encourage them to share their thoughts with a medical or mental health professional.** There are numerous possible situations where this expertise is needed, so don’t hesitate to reach out. Ultimately, you want to help keep them safe.

*Disclaimer: This document is a general educational resource on helping people with illness anxiety disorder diagnosed by a physician. The guidelines here may not apply to every case. Healthcare providers who distribute this to staff, patients and families are responsible for ensuring the material presented here is accurate and appropriate for their patients’ care. This guide does not constitute a medical or psychological treatment.*

# References

First, M.B., Skodol, A.E., Williams, J.B.W., Spitzer, R.L. (2016). “Illness Anxiety Disorder,” in Learning DSM-5® by Case Example, Washington, DC: American Psychiatric Association Publishing, 200-202.

Tyrer, P. (2018). Recent advances in the understanding and treatment of health anxiety. Current Psychiatry Reports, 20(7), 49. doi: 10.1007/s11920-018-0912-0

Witthöft, M., Hiller, W. (2010). Psychological approaches to origins and treatments of somatoform disorders. Annual Review of Clinical Psychology, 6, 257-283. doi: 10.1146/annurev.clinpsy.121208.131505
